# Recent Advances on Pt-Based Compounds for Theranostic Applications

**DOI:** 10.3390/molecules29153453

**Published:** 2024-07-23

**Authors:** Giulia Ferrari, Ines Lopez-Martinez, Thomas Wanek, Claudia Kuntner, Diego Montagner

**Affiliations:** 1 Department of Chemistry, Maynooth University, W23 F2H6 Maynooth, Ireland; 2 Division of Nuclear Medicine, Department of Biomedical Imaging and Image–Guided Therapy, Medical University of Vienna, 1090 Vienna, Austria; 3 Preclinical Imaging Lab (PIL), Department of Biomedical Imaging and Image–Guided Therapy, Medical University of Vienna, 1090 Vienna, Austria; thomas.wanek@meduniwien.ac.at (T.W.); claudia.kuntner@meduniwien.ac.at (C.K.); 4 Medical Imaging Cluster (MIC), Medical University of Vienna, 1090 Vienna, Austria; 5 Kathleen Londsdale Institute for Human Health Research, Maynooth University, W23 F2H6 Maynooth, Ireland

**Keywords:** platinum chemotherapy, theranostic, molecular imaging, cancer therapy, therapy monitoring

## Abstract

Since the discovery of cisplatin’s antitumoral activity and its approval as an anticancer drug, significant efforts have been made to enhance its physiological stability and anticancer efficacy and to reduce its side effects. With the rapid development of targeted and personalized therapies, and the promising theranostic approach, platinum drugs have found new opportunities in more sophisticated systems. Theranostic agents combine diagnostic and therapeutic moieties in one scaffold, enabling simultaneous disease monitoring, therapy delivery, response tracking, and treatment efficacy evaluation. In these systems, the platinum core serves as the therapeutic agent, while the functionalized ligand provides diagnostic tools using various imaging techniques. This review aims to highlight the significant role of platinum–based complexes in theranostic applications, and, to the best of our knowledge, this is the first focused contribution on this type of platinum compounds. This review presents a brief introduction to the development of platinum chemotherapeutic drugs, their limitations, and resistance mechanisms. It then describes recent advancements in integrating platinum complexes with diagnostic agents for both tumor treatment and monitoring. The main body is organized into three categories based on imaging techniques: fluorescence, positron emission tomography (PET), single–photon emission computed tomography (SPECT), and magnetic resonance imaging (MRI). Finally, this review outlines promising strategies and future perspectives in this evolving field.

## 1. Introduction

Cis-dichlorodiammine platinum(II), commonly known as cisplatin, is one of the most known and worldwide used chemotherapy agent for the treatment of several kinds of cancers in adults. The fortunate and accidental discovery of anticancer properties in 1965 by B. Rosenberg, followed by the Food and Drug Administration (FDA) approval (1978), triggered extensive research in this field, leading to the development of novel therapeutics based on a metallic scaffold. As a result, platinum(II) complexes have emerged as the most successful category of metal–based anticancer drugs, receiving enormous attention for over half a century [1,2].

Unfortunately, the use of cisplatin is limited due to severe dose-limiting side effects which arise from the drug’s indiscriminate uptake by all rapidly dividing cells, coupled with the body’s attempt to eliminate the exogenous drug through the kidneys. These adverse effects include nephrotoxicity, neurotoxicity, ototoxicity, myelosuppression, intrinsic/acquired therapy resistance, and the inconvenient intravenous administration route [3,4].

### 1.1. Mechanism of Action of Cisplatin and Resistance Mechanisms

**Figure 1:**
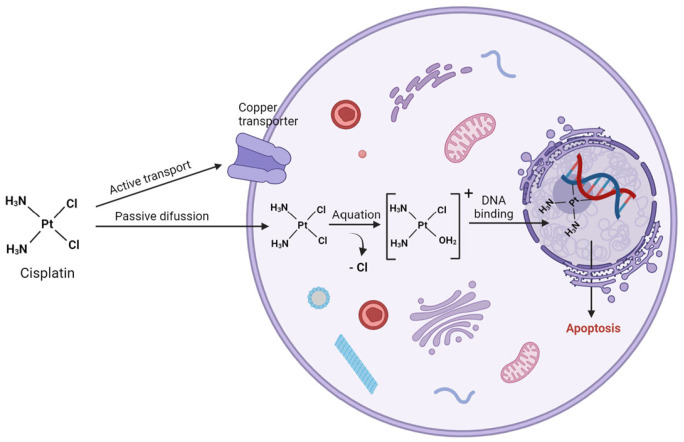
Mechanism of action of cisplatin (created with BioRender.com).

The action mechanism of cisplatin is nowadays well known (Figure 1). Cellular absorption relies on a combination of passive diffusion through the plasma membrane and active transport facilitated by membrane proteins, specifically copper transporter ions and organic cation transporters. Notably, transmembrane copper transport protein 1 (CTR1) is involved in copper homeostasis and cisplatin uptake. Indeed, downregulation of CTR1 is frequently observed in cisplatin-resistant cells, contributing to poor therapeutic responses in non–small cell lung cancer (NSCLC) patients [5].

On the other hand, copper-extruding P-type ATPases (ATP7A and ATP7B) regulate cisplatin export [6]. These transporters are observed to be upregulated in cisplatin-resistant cancer cells and high levels of ATP7A and ATP7B are associated with significantly poorer overall survival in patients with ovarian cancer and ovarian carcinoma, respectively [7,8]. Following intravenous administration of cisplatin, its intracellular activation relies on the drug’s high tendency to undergo aquation where one or both chloride ligand(s) are replaced by one or two molecules of water. This reaction is facilitated by the square planar geometry of cisplatin and is further promoted by the lower chloride concentration in the cytoplasm (4–20 mM) compared to the bloodstream (100 mM). These reactive cisplatin species (mono-aquo and di-aquo complexes) are then prone to bind to cytoplasmic S-containing nucleophiles, such as glutathione (GSH), metallothioneins and other cysteine-rich proteins. Indeed, high levels of glutathione, glutathione-S-transferase or γ-glutamyl cysteine synthetase have been observed in cisplatin-resistant cells [9,10,11]. The activated platinum complexes, carrying positive charges, will also establish interactions with negatively charged DNA. Through this mechanism, cisplatin covalently binds preferentially to the N7 position of guanine nucleobase and to a lesser extent, adenine. Several DNA-Pt adducts have been identified, with the major ones being the cross-links between two adjacent guanines, (intrastrand DNA) and between two guanine bases from different DNA strands (interstrand adducts). These adducts induce a distortion in the DNA structure ranging from 10 to 60 degrees towards the major groove and unwind the helix by up to 23 degrees [12,13], hindering both replication and transcription, ultimately leading to apoptotic cell death. Several protein families are involved in the recognition of Pt-DNA adducts that finally lead to cisplatin-resistant mechanisms, including nucleotide excision repair (NER) proteins, mismatch repair (MMR) proteins and non-histone chromosomal high-mobility group protein 1 (HMGB1). Cisplatin resistance mechanisms can be classified as pre-target (alterations in cisplatin transport before DNA binding), on-target (repair of Pt-DNA lesions), post-target (cellular events occurring after formation of DNA-Pt adducts) and off-target (alterations in signaling pathways indirectly hindering cisplatin-induced proapoptotic events) [14]. Table 1 reports the major resistance mechanisms associated with cisplatin chemotherapy.

To circumvent clinical cisplatin resistance and side effects, several platinum analogues with diverse structural motifs have been developed and explored for their antitumor properties. While some exhibit promising in vitro and in vivo biological activity, the translation to clinic for most of these species has been hindered by various challenges [1].

**Figure 2:**
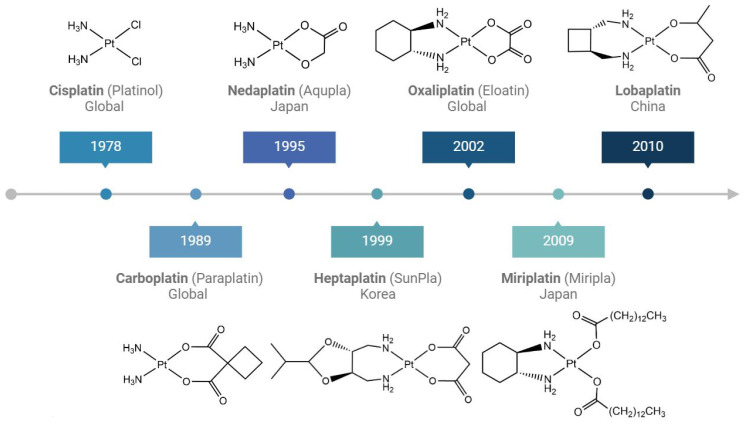
Platinum drugs in clinical use with their respective approval years.

Besides cisplatin, two more platinum(II)-based anticancer drugs received approval by FDA, carboplatin and oxaliplatin, called the “second generation of Pt chemotherapeutics”, which showed a higher physiological stability due to the chelate ligand(s) (Figure 2) [36]. In addition, four more platinum(II) complexes have received approval for human use (Figure 2). Nedaplatin, a second-generation drug, has been approved in Japan for the treatment of NSCLC, small cell lung cancer (SCLC), esophageal cancer, and head and neck cancers. It demonstrates higher activity than carboplatin and comparable efficacy to cisplatin. Heptaplatin is approved in South Korea for the treatment of gastric cancer. More recently, in 2009, Miriplatin was approved in Japan for treating hepatocellular carcinoma [37,38], and Lobaplatin was approved in China for the treatment of chronic myelogenous leukemia, inoperable metastatic breast cancer and SCLC. Most of the previously mentioned drugs offer advantages over cisplatin, such as drug resistance alleviation, toxicity and side effects mitigation, and water solubility improvement.

### 1.2. Pt(IV) Complexes to Improve Cytotoxicity and to Overcome Resistance

**Figure 3:**
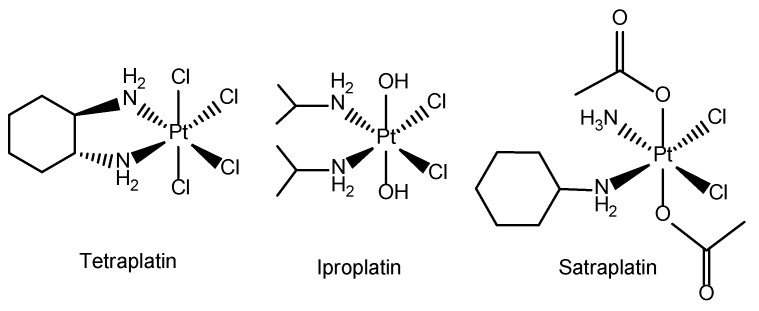
Structure of Pt(IV) compounds tetraplatin, iproplatin and satraplatin.

In the last two decades, a lot of scientific interest has moved toward the synthesis and application of Pt(IV) species. These complexes are prodrugs because they must be intracellularly activated by reduction, releasing the active Pt(II) scaffold and restoring their latent cytotoxic activity [39]. Pt(IV) complexes are prepared by oxidative addition of the square planar Pt(II) complexes to yield the octahedral complex while retaining the original Pt(II) equatorial coordination sphere. The major advantage of Pt(IV) species is their higher stability and lower reactivity toward S-containing nucleophiles, as a result of the octahedral geometry (d^6^ low-spin electronic configuration) compared to the square planar Pt(II) counterparts (d^8^). The combination of two extra axial ligands to fine-tune chemical properties and high kinetic inertness to substitution reactions have turned Pt(IV) complexes into promising anticancer drug candidates. To date, three Pt(IV) compounds (tetraplatin, iproplatin and satraplatin) have undergone clinical trials, but none have gained clinical approval yet (Figure 3). Tetraplatin was abandoned due to severe neurotoxicity, while iproplatin demonstrated lower activity compared with carboplatin and cisplatin. Satraplatin, the first orally administered platinum(IV) complex, exhibited documented efficacy and acceptable safety in patients with hormone-refractory prostate cancer and small-cell lung cancer. Despite this, it failed to gain FDA approval due to the low convincing benefits in terms of overall survival [4,40].

The axial ligands of the Pt(IV) complexes can be viewed as cargos that the cytotoxic Pt(II) moiety unloads inside the cell. These ligands can play various roles, serving as passive uptake enhancers (lipophilic moieties) on cancer cells or subcellular targeting agents. Additionally, they can be designed to tether the prodrug to delivery system like polymers or nanoparticles. They can also be bioactive moieties such as other drugs, enzyme inhibitors, pathway activators or suppressor, epigenetic modifiers, and antimetabolites. The integration of these ligands aims to synergistically enhance the pharmacological properties in conjunction with the Pt(II) moiety [2].

### 1.3. Translation to Theranostics

Besides the therapeutic properties, an appropriate functionalization of the Pt-based species can play an important role in the diagnostic detection of cancer. Through modification with biocompatible nanomaterials or appropriate functional ligands, the therapeutic units may have the potential to evolve into theranostic agents, offering simultaneously therapeutic and diagnostic applications [41].

The term “theranostic” (syn: theragnostic) has been coined to describe platforms that serve dual roles as diagnostic and therapeutic agents. A broader definition has also been proposed, referring to an integrated therapeutic system, which can diagnose, deliver targeted therapy, and track the response to therapy. Indeed, the idea is that theranostic agents could provide the ability to simultaneously monitor the disease and the efficacy of the treatment [42]. This combination promises to create a paradigm shift in individual patient care through an appropriate diagnostic methodology with an optimized treatment strategy to personalize a separate therapeutic intervention [43].

This emerging field in personalized medicine is guided by ligands targeting malfunctioning cells. In the realm of cancer theranostics, diverse strategies involving nanomaterials, metal complexes or small molecules have been developed. As an example, metallic, lipidic and polymer nanoparticles exhibit a variety of biological characteristics that can be used for theranostic purposes. While clinical approval for the use of nanomaterials as transport vehicles has been longstanding, clinical translation of nanomaterial-based theranostic systems from bench to bedside has faced major hurdles [44].

In this review, our primary focus lies in exploring different strategies for the integration of Pt complexes with diagnostic probes to obtain theranostic systems. According to the design goals, we divided them into three categories depending on the main imaging techniques used in combination with the anticancer drug: (i) optical imaging (fluorescence), (ii) positron emission tomography (PET) and single-photon emission computed tomography (SPECT) and (iii) magnetic resonance imaging (MRI). The applications, advantages, and disadvantages of these commonly used imaging techniques are summarized in Table 2. Every imaging modality will be discussed in more detail in the subsequent sections and their application as Pt-based theranostic agents.

## 2. Illuminating Disease through Fluorescence Imaging

**Figure 4:**
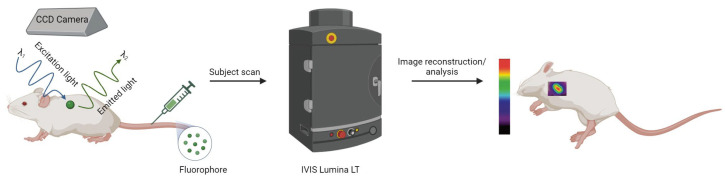
Basic principles of optical fluorescence molecular imaging. Following administration of the fluorophore, the subject is illuminated with excitation light (λ_1_). This excites the fluorophore, causing it to emit light (λ_2_), which is then captured by a CCD camera within IVIS Lumina LT in vivo imaging system, processed, and converted into an image (created with BioRender.com).

Fluorescence imaging is a non-invasive technique that allows for the monitoring of the dynamics of luminescent molecules within cells or small animals, offering high sensitivity and excellent temporal resolution. A targeted biological compound is exposed to a specific radiation (absorption) and the subsequent emitted light (emission) is captured for analysis. Utilizing standard infrastructure like a charged-couple device (CCD) camera and a light source, fluorescence imaging proves to be a cost-effective method (Figure 4) [47]. Additionally, its remarkable sensitivity capable of detecting concentrations as low as picomolar to femtomolar levels enables the visualization of individual molecules and facilitates the tracking of biological processes. Unlike imaging techniques involving the detection of high-energy gamma rays such as PET and SPECT, fluorescence imaging operates with low-energy photons, ensuring its safety for both samples and operators. However, a significant limitation of this technique lies in its restricted tissue penetration, thereby impeding optical imaging of probes in deep tissues or large subjects, consequently hindering its clinical translation [48].

A persisting challenge when studying Pt-based drugs in vitro and/or in vivo is that there are a few spectroscopic techniques available for detecting their faith. The main techniques employed to measure and quantify the cellular internalization of Pt-based samples in cells are Graphite Furnace Atomic Absorption Spectrometry (GFAAS) and Inductively Coupled Plasma Mass Spectrometry (ICP-MS) analysis [49,50] but, unfortunately, no information regarding speciation and cellular localization could be obtained. To address these challenges, several Pt complexes containing tethered fluorophores have been generated to evaluate its pharmacokinetic behavior. This assessment covers organ accumulation, systemic circulation time, intracellular target, and excretion patterns.

**Figure 5:**
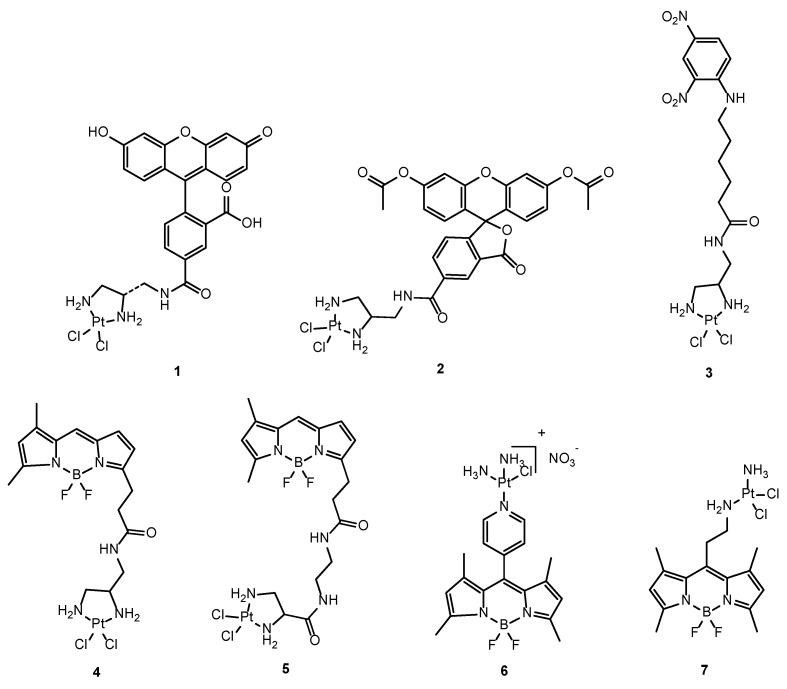
Chemical structures of the different fluorophore-cisplatin analogues: FDDP (**1**), CFDA-Pt (**2**), DNP-Pt (**3**), BODIPY-Pt (**4**–**6**), CP-11 (**7**).

Most of these compounds retained the traditional structure–activity requirements for Pt(II) anticancer compounds (square planar complexes with leaving groups and amines in *cis* orientation) [51]. Initial investigations involved linking cisplatin with fluorophores such as fluorescein (**1**) [52] carboxyfluorescein diacetate (CFDA), (**2**), 6-(2,4-dinitrophenyl)aminohexanoic acid (DNP), (**3**) [53], and Alexa [54] (Figure 5). These complexes have been used to monitor the distribution of cisplatin in human ovarian carcinoma, human osteosarcoma, and human cervical carcinoma cells, respectively. Remarkably, they demonstrated swift accumulation of fluorophore-cisplatin analogues in the nucleus, succeeded by sequestration within Golgi vesicles. More recent works described a series of Pt-based complexes tethered to boron-dipyrromethene (BODIPY) [55,56,57] (Figure 5). This family of fluorophores has gained considerable interest in bioimaging because of their high absorption coefficients, sharp emission, high fluorescence quantum yields and excellent chemical and photostability. Both **4** and **5** showed primarily cytosolic localization, with nuclear distribution observed only at higher concentrations [55]. Regarding compound **6**, colocalization experiments with commercially available mitochondria-specific dye (MitoTracker) revealed its predominantly localization in the mitochondria, while nuclear colocalization remained indiscernible [56]. Moreover, in vivo pharmacokinetics studies of these compounds have also been conducted. Using a window chamber tumor model, which enables simultaneous time-course fluorescence microscopy of the fluorescent drug in both mouse vasculature and tumor tissue, **7** showed plasma kinetics comparable to cisplatin. Furthermore, high-resolution intravital imaging was employed to monitor DNA damage in individual tumor cells following administration. The study focused on the fluorescence signal of 53BP1 puncta, which are markers of DNA damage response within the cells. No linear correlation was observed between the fluorescence signal of these 53BP1 puncta and the accumulation of the drug CP11, indicating potential heterogeneity in the cells’ drug response [57].

Nevertheless, incorporating a fluorophore into a small inorganic complex can significantly modify its physicochemical properties and biological activity. Such an approach must consider the organelle selectivity of the fluorophore, which may vary depending on parameters such as molecular weight, partition coefficient, amphiphilic character and pK_a_ values [58]. Thus, it is uncertain if fluorescent analogues accurately model cisplatin’s cellular behavior, and, likewise, bulky fluorophores may interfere with DNA binding mechanisms of platinum. Furthermore, some of them, and in particular BODIPY, can generate ROS (Reactive Oxygen Species) under irradiation that can potentially interfere with the cell damage mechanism. Notably, none of the previous Pt-fluorophore complexes showed significant nuclear localization.

**Figure 6:**
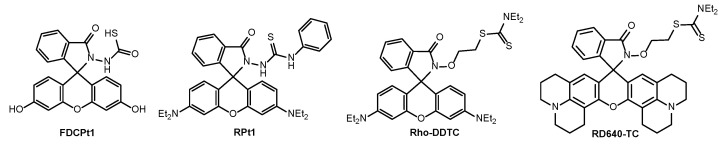
Chemical structures of different exogeneous fluorescent probes.

To address these limitations and to allow for a “label-free” study of Pt drug fate, several exogeneous fluorophores have been synthesized to investigate the speciation of platinum anticancer drugs. For example, FCDPt1 (Figure 6) is a fluorescein-based probe linked to dithiocarbamic acid moiety which selectively identifies platinum species [59]. Similarly, RPt1, a rhodamine-based probe incorporating phenyl isothiocyanate, has been developed for studying metabolism of trans-Pt complexes [60]. Furthermore, Rho-DDTC was the first rhodamine-based fluorescent turn-on probe designed to distinguish between Pt(II) and Pt(IV) complexes upon intracellular reduction [61,62]. Following this discovery, numerous analogues were developed to enhance fluorescence intensity and sensitivity of these probe [63,64]. Probe RD640-TC was developed as the first tool for in vivo tracking of cisplatin due to its remarkable characteristics such as high fluorescence quantum yield (Φ = 0.68), two-photon absorption properties (enabling greater penetration depth) and high photostability (Figure 6) [65].

**Figure 7:**
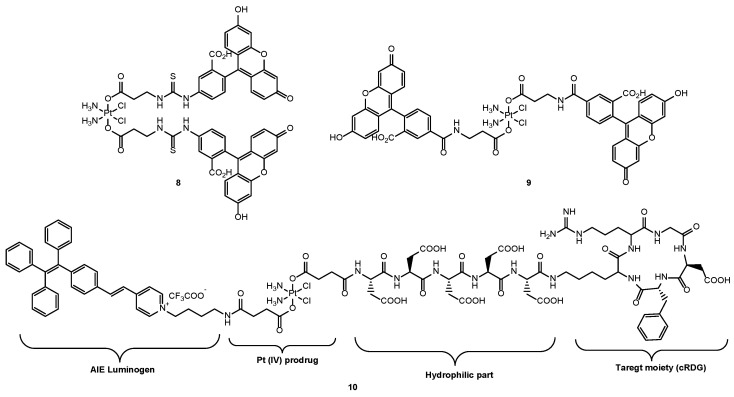
Chemical structures of the different Pt(IV)-fluorescent scaffolds: Pt(IV) (FITC)_2_ (**8**), Pt(IV) FL_2_ (**9**), Pt(IV) prodrug-AIE conjugate (**10**).

More examples of turn-on probes include the work by Lippard et al. [66], who synthesized two analogues of fluorescein conjugated to cisplatin Pt(IV) prodrugs, **8** and **9**, and that of Bin Liu et al. [39], who developed a cisplatin Pt(IV) prodrug conjugated to an aggregation-induced emission (AIE) luminogen (**10**). In both cases, the reduction of Pt(IV) to Pt(II) within the cellular environment was monitored by the release of fluorescein ligands and luminogen, respectively. Additionally, Bin Liu et al. incorporated a short hydrophilic peptide to ensure water solubility and a cyclic arginine-glycine-aspartic acid (cRGD) tripeptide as a targeting ligand (Figure 7).

To promote the clinical translation of fluorophores, both near infrared NIR-I (700–900 nm) and NIR-II dyes (1000–1700 nm) have emerged as imaging tools to afford precise dynamic actions in vivo with high spatiotemporal resolution, deeper penetration and decreasing light absorption and scattering (Table 3) [67,68].

**Figure 8:**
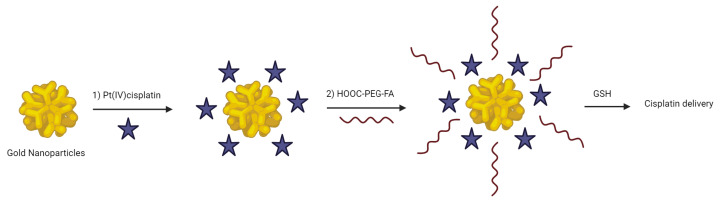
Schematic illustration of AuNCs-based theranostic platform. The star symbol corresponds to Pt(IV)-cisplatin based scaffold and the wavy lines to HOOC–PEG–FA compound (created with BioRender.com).

Among these, gold nanoclusters (AuNCs) stand out for their remarkable properties: excellent biocompatibility, size below the renal excretion threshold, robust photostability, facile modification, and excellent photothermal activity [69]. In a study conducted by Li et al., AuNCs were coupled with Pt(IV)-cisplatin and folic acid (FA), enabling not only fluorescence imaging but also targeted chemotherapy for breast cancer (Figure 8). Biodistribution analysis revealed that FA-modified AuNC-Pt nanoparticles demonstrated high tumor uptake, effectively evaded reticuloendothelial system (RES) capture, and facilitated renal clearance [70].

Similar studies were carried out by Zhiqiang Yu et al., who reported a cisplatin delivery platform based on AuNCs. The results showed that not only AuNCs-Pt does effectively bind glutathione (GSH) via Au-S bonds, scavenging intracellular GSH and thereby facilitating increased internalization of cisplatin, leading to enhanced anticancer activity, but also that the unique NIR-II imaging capability of AuNCs-Pt enables precise visualization of Pt transport in both ovarian and hepatocellular carcinoma tumor models. Importantly, in vivo NIR-II imaging revealed that AuNCs-Pt exhibited significantly superior tumor growth inhibition compared to cisplatin while inducing minimal systemic toxicity, underscoring its potential as a highly effective theranostic agent [71].

Another class of NIR-I fluorescent probes are conjugated polymers, which are promising for their high brightness and low toxicity. Bin Liu et al. employed conjugated polyelectrolytes (CPE) grafted with polyethylene glycol (PEG) chains to load cisplatin for targeted in vitro and in vivo NIR imaging. These nanoparticles enable sustained cisplatin release, enhancing internalization. While these nanoparticles hold potential for liver cancer applications due to their high accumulation in RES, further optimization of their physical and chemical properties is needed for broader cancer imaging and chemotherapy applications [72].

**Figure 9:**
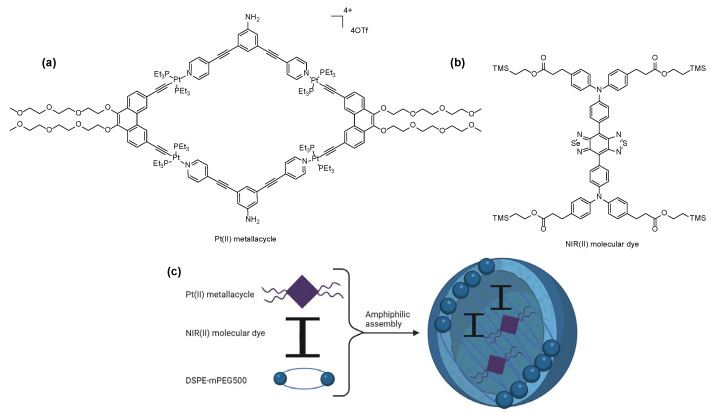
Structures of (**a**) Pt(II)metallacycle, (**b**) NIR(II) molecular dye and (**c**) representation of the nanoplatform (created with BioRender.com).

It has been well known that Pt(II) metallacycles-based supramolecular coordination complexes can act as anticancer agents; however, poor photostability, low tumor uptake and penetration depth limited the in vivo antitumor applications [73]. Recently, Stang et al. designed a NIR-II theranostic nanoprobe that incorporates a Pt(II) metallacycle and an organic molecular dye (benzobisthiadiazole) into DSPE-mPEG500 to enhance stability and biocompatibility of the theranostic agent in vivo (Figure 9). Compared to cisplatin, this NIR-II theranostic agent displayed better efficiency of tumor growth inhibition and prolonged blood circulation, as well as reduced systemic toxicity [74].

In a parallel investigation, Han et al. employed a benzobisthidiazole-based NIR II imaging probe (TQTPA) to synthesize multimodal nanoparticles loaded with both TQTPA and cisplatin using hyaluronic acid, enabling active targeting and enhanced accumulation in tumor tissue. The therapeutic efficacy of this chemo-photothermal synergistic approach was assessed in a mouse model. Upon irradiation, the tumor site temperatures significantly exceeded those of the negative control. Moreover, after 13 days of treatment, tumor volume decreased by 83% compared to the untreated control group [75].

**Figure 10:**
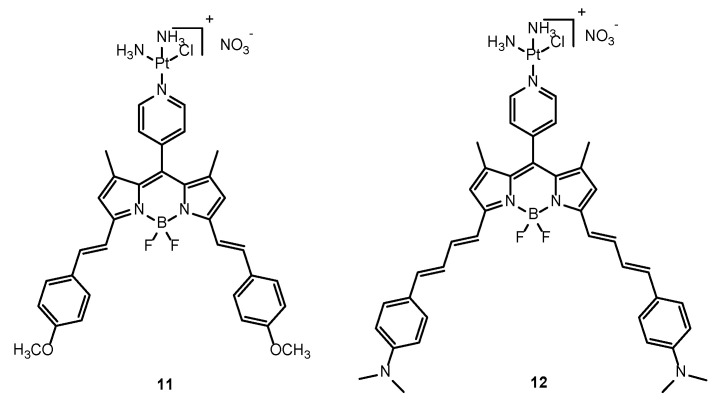
Chemical structures of BODIPY-based NIR imaging probes (**11** and **12**).

BODIPY fluorophores can also be used for NIR imaging by implementing π-conjugated structures (red-shifted absorbance), (Figure 10). In this way, Zhigang Xie et al. synthesized compound **11** (λ_abs_ = 645 nm, λ_em_ = 674 nm), demonstrating its internalization in HepG2 cells and localization within mitochondria. Through MTT assays, **11** showed comparable cytotoxicity to cisplatin in HepG2 and HeLa cells. Through NIR imaging, **11** was localized at tumor sites four hours post-injection [76]. Following this work, Minhuan Lan et al. further enhanced NIR imaging capabilities by synthesizing a NIR cisplatin-appended BODIPY fluorophore (**12**) with greater red-shifted absorption (λ_abs_ = 748 nm, λ_em_ = 947 nm) through the incorporation of N,N-dimethylaniline into BODIPY (Figure 10) [77].

Quantum dots (QDs) are renowned for their distinct optical features like good photostability, large Stokes shift, and high fluorescence quantum yields. In a study by Rylander et al., a novel hybrid system was developed, comprising self-assembled single-walled carbon nanohorns (SWNHs) loaded with cisplatin and adorned with CdSe/ZnS quantum dots on their exterior. Through fluorescence imaging, SWNHs–QD+cis hybrids were tracked in rat bladder transitional carcinoma cells after 72 h incubation. These images helped to validate the mechanism of continued cell death over 72 h as well as the compound’s internalization and cisplatin delivery on the nucleus over time [78]. However, a significant drawback of metal-based quantum dots lies in their inherent toxicity. Indeed, the previous work assessed cell viability using SWNHs as a negative control but did not include evaluation with SWNHs+QDs. Consequently, graphene quantum dots (GQDs) have emerged as alternative, non-toxic nanocarriers with superior biocompatibility and fluorescent properties. As an example, Sierin Lim et al. designed GQDs functionalized with anti-epidermal growth factor receptor antibodies and loaded with cisplatin, achieving targeted delivery of cisplatin in breast cancer cells, as validated through fluorescence imaging [79]. Moreover, GQDs offer the possibility to fine-tune their optical properties, enabling the development of NIR-II imaging QDs for in vivo NIR imaging-guided photothermal therapy [80]. This platform could also be coupled with cisplatin/Pt(IV) prodrugs delivery for multimodal therapeutic applications. Table 3 highlights the advantages, disadvantages, applications and future research on the NIR-fluorophores mentioned in this section [81,82].

**Table 3:** Summary of the advantages, disadvantages, applications and future research on the NIR-fluorophores mentioned in this chapter [81,82].

NIR Fluorophores	Advantages	Disadvantages	Applications	Future Research
AuNCs	Easy surface modification. High biocompatibility. Large Stokes shift.	Photobleaching.	Bioimaging and therapeutic applications.	Escaping recognition by immune system.
Conjugated polymers	High brightness. Low toxicity.	Limitation of excitation and emission wavelengths (<900 nm).	Photoacoustic imaging.	Increase polymer’s conjugation degree to shift spectra towards NIR-II window.
Benzobisthiadizole	Large Stokes shifts. High imaging quality.	Photobleaching.	Probes for clinical translation.	Design of new materials for NIR-II imaging.
BODIPYs	High quantum yields. Excellent photostability.	Poor water solubility.	In vivo visualization of tumors.	Reducing bandgap of fluorophores to bathochromic shift wavelength enhancing hydrolytic stability.
QDs	High quantum yield. Good photostability.	Toxicity.	In vitro evaluation.	Design of non-toxic QDs for in vivo NIR imaging.

## 3. Pioneering Platinum-Radiopharmaceuticals for SPECT and PET Imaging

**Figure 11:**
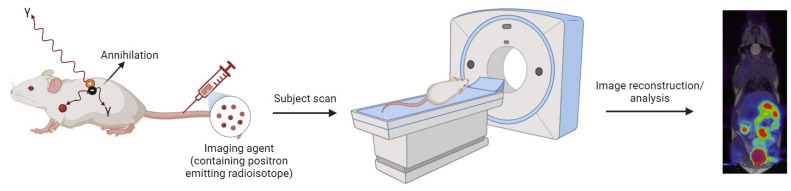
Schematic representation of the basic principles of PET (created with BioRender.com).

SPECT/PET are radionuclide molecular imaging modalities that allow for the real-time monitoring of drugs’ distribution within the body as well as non-invasive monitoring of their therapeutic efficacy [83]. The fundamental principle of SPECT is the administering of a radiotracer-containing imaging agent (such as a peptide, protein, or nanoparticle) with a gamma-emitting radioisotope (Technetium-99m, Iodine-123, Thallium-201) to the patient. Subsequently, gamma rays emitted by the tracer are captured by a rotating gamma camera, which collects data from various angles to facilitate tomographic reconstruction. On the other hand, PET employs positron-emitting radioisotopes, such as fluorine-18, Gallium-68, Copper-64, Zirconium-89. Positrons emitted by the tracer will then collide with electrons in the surrounding tissues, a phenomenon known as annihilation, which results in the creation of two gamma rays, each with an energy of 511 keV, moving in opposite directions. The PET detector detects these gamma rays which determine the location of annihilation event. The resulting electrical signals are then converted into sinograms, which are further reconstructed into detailed tomographic images (Figure 11) [47]. While both techniques are similar, PET exhibits significantly higher sensitivity, typically ranging from two to three orders of magnitude greater than that of SPECT [84].

**Figure 12:**
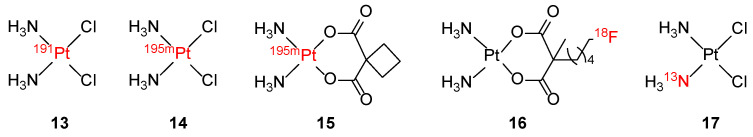
Chemical structures of ^191^Pt-CDDP (**13**), ^195m^Pt-CDDP (CISSPECT^®^), (**14**), ^195m^Pt-CP (**15**), ^18^F-FCP (**16**) and ^13^N-CDDP (**17**).

The development of smart molecules, possessing both therapeutic and diagnostic properties, holds the potential to revolutionize cancer therapy by enhancing efficiency and safety. The simplest examples of such theranostics are the radioactive forms of cisplatin (**CDDP**) and carboplatin (**CP**) such as ^191^Pt/^195m^Pt-CDDP, ^195m^Pt-CP, ^18^F-FCP (a derivative of carboplatin) and ^13^N-CDDP (Figure 12).

Among the four radioactive isotopes of Pt (Platinum-191, Platinum-193m, Platinum-195m, Platinum-197), Platinum-195m has gained special attention over the years due to its ease of production, ideal gamma energy spectrum (similar toThallium-201) and appropriate half-life (4 days) for efficient synthesis, quality control and in vivo radio-imaging (Table 4).

The synthesis of Platinum-195m has been well established [85,86], and relies on the thermal neutron irradiation of ^194^Pt with yields ranging from 60 to 90%. More recently, an alternative preparation method has been introduced to enhance the specific activity of Platinum-195m. This method involves irradiating ^197^Au with breaking radiation. Notably, this novel approach has demonstrated a remarkable 37,000-fold increase in specific activity over a span of 25 years [87].

Platinum-195m is not a pure gamma emitter, as each disintegration also releases 36 auger electrons that deposit large amounts of energy (around 25 keV) within nanometer-micrometer distances in tissues. Therefore, integration of cisplatin to Platinum-195m emerges as a versatile platform, offering the potential for both enhanced therapeutic effects and tumor imaging. For this reason, several studies were conducted with **14**, also known as (CISSPECT^®^). Using **14** and **15**, Wolf et al. studied non-invasive in vivo pharmacokinetics of these compounds in Walker 526 solid tumors bearing rats [88]. In a more recent study, **14** proved to be a safe radiopharmaceutical, highlighting its potential for optimizing dosage in patients undergoing cisplatin chemotherapy. Moreover, the radiopharmaceutical presented a favorable biodistribution profile in which the kidneys received the highest dose accounting for a clearance half-life of 40 h [89]. Later, the first preclinical SPECT images of **14** were conducted, showing its potential as a tracer to image the biodistribution of platinum-based compounds in vivo [90]. In addition, **14** has proven to be more effective than regular cisplatin due to its enhanced toxicity after incubation in the Chinese hamster V79 cell line [91]. Its enhanced toxicity needs to be further validated in in vivo models [92].

**Figure 13:**
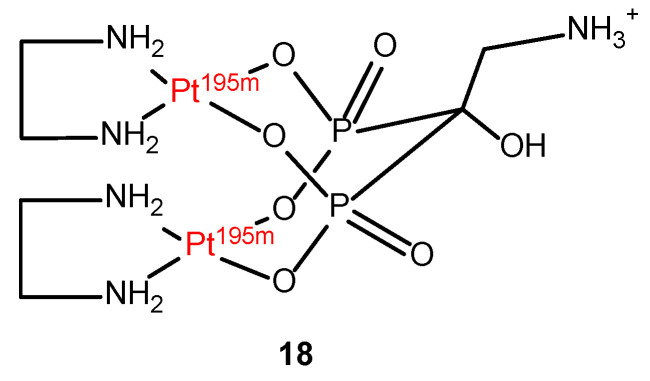
Chemical structure of ^195m^Pt-biphosphonate (^195m^Pt-BP) compound (**18**).

Given the inherent therapeutic efficacy of the Platinum-195m radioisotope, there is a current trend in the development of radio-therapeutically active compounds. An illustrative example is the development of ^195m^Pt-biphosphonates (BP) complex **18** (Figure 13). The bisphosphonate ligand was chosen to specifically target bone cancers due to high affinity for Ca^2+^.

This complex (**18**) has demonstrated a specific accumulation in bone sites exhibiting high metabolic activity, resulting in an impressive 11-fold increase in DNA damage within metastatic tumor cells when compared to non-radioactive Pt-BP. Furthermore, the biodistribution of these compounds can be precisely monitored through SPECT imaging, revealing minimal systemic toxicity. This opens a promising avenue for further research in the field of radiopharmaceuticals, particularly for the treatment of bone cancer [93,94]. On the other hand, earlier studies used **13** to examine biodistribution and kinetics in patients undergoing cisplatin treatment [95,96], following the demonstration of its enhanced antitumor effect in both in vitro and in vivo models compared to non-radioactive cisplatin [97,98,99].

However, the further application of this radioisotope for human imaging studies is hindered by certain drawbacks. Above all, its high energy γ-photons (539 keV) are not ideal for high-contrast radio imaging using modern gamma cameras. Moreover, unlike Platinum-195m, which decays to ^195^Pt, Platinum-191 decays to ^191^Ir which is a different element with different pharmacokinetics leading to regulatory challenges in human imaging studies. Little research has been carried out regarding Platinum-193m and ^197^Platinum-197 radioisotopes [100].

The synthesis of **17** has also been reported through several methods [101,102] to investigate the pharmacokinetics of cisplatin in both mice and brain tumor patients [103]. However, its clinical use is constrained by its very short half-life (t_1/2_ = 10 min).

A different approach was taken by Zweit et al., who developed compound **16**, a feasible PET tracer for assessing the biodistribution of carboplatin [104]. Fluorine-18 is widely used and is the preferred radioisotope for PET imaging due to its favorable characteristics such as half-life of 109 min, high positron emission (97%), suitable positron energy (0.634 MeV) and being easily produced by cyclotron (Table 5) [105].

Compared to Platinum-195m, Fluorine-18 offers several advantages for clinical use. The routine production and supply of Platinum-195m face significant challenges due to its high cost and logistical difficulties. On the other hand, ^18^F has high availability, versatile chemistry, and compatibility with a wide range of biomolecules.

In this way, **16** demonstrated its capability to assess the drug distribution across various tumor types, showcasing its potential to enhance the overall efficacy of platinum-based chemotherapy on an individual patient basis. Following this discovery, a versatile platform consisting of **16** encapsulated in ^111^In-labeled liposomes was synthesized for image-guided drug delivery [107]. This study proves the dual-tracer imaging feasibility of SPECT and PET probes in vivo, aiming to enhance the precision of drug tracing within the body.

In more recent studies, supramolecular structures were developed that can encapsulate Pt drugs while incorporating radionuclides for in vivo PET imaging. An example is the work by Casini et al. [108], who developed a palladium-based metallo-cage scaffold to encapsulate cisplatin while incorporating Fluorine-18. Although very promising outcomes were obtained, further investigation is needed to refine the kinetic stability of these supramolecular structures, ensuring optimal biodistribution profiles for future clinical applications. Despite the synthesis of numerous Pt(II)-metallo-assemblies, their potential as imaging-assisted cancer diagnosis agents is still in an early stage of exploration [109].

**Figure 14:**
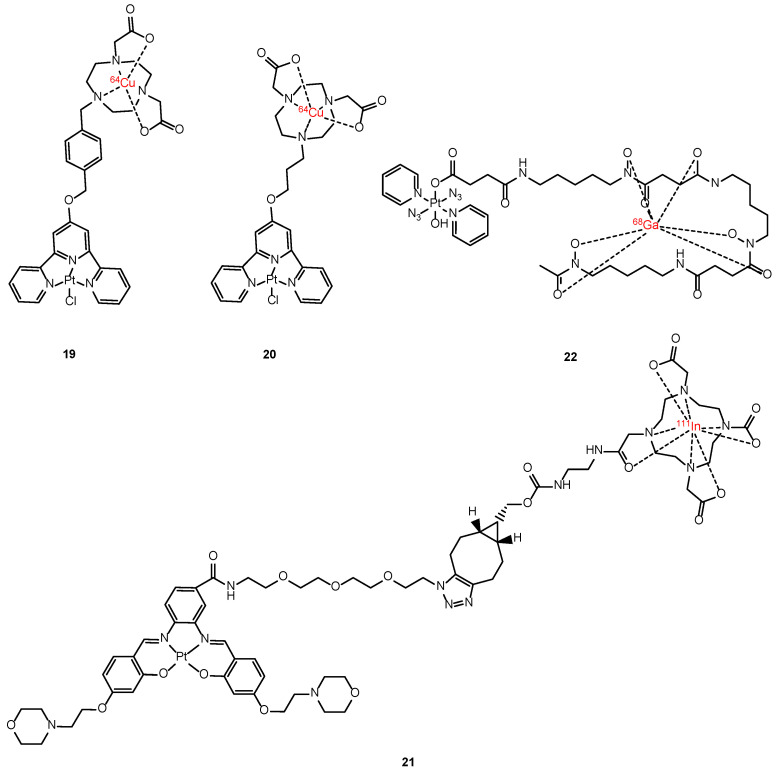
Chemical structures of [^64^Cu]Cu-NOTA-TP (**19**), [^64^Cu]Cu-NOTA-C3-TP (**20**), Pt(II)salpen-^111^In (**21**) and Pt-succ-DFO-^68^Ga (**22**).

Terpyridine (TP) platinum-based complexes have been developed as theranostic platforms with a specific focus on targeting G-quadruplexes to overcome platinum drug resistance (Figure 14). These complexes are conjugated with ^64^Cu-NOTA (NOTA = 1,4,7-Triazacyclononane-1,4,7-triacetic acid) for targeted radiotherapy. This radioisotope (t_1/2_ = 12.8 h; EC, [43.1%], β+, 0.653 MeV [18%]; β-, 0.579 MeV [38.4%]) not only possesses PET imaging properties via β+ emission but also holds potential for cancer therapy via auger electron emission. Guérin et al. synthesized two ^64^Cu-NOTA-TP conjugates (**19** and **20**), each featuring a unique TP-NOTA linker, with the aim of investigating their cytotoxic effects in both human colorectal tumor cells and a normal fibroblast cell line (Figure 14). Notably, the use of a flexible linker in **20** demonstrated superior selectivity towards cancer cells, a higher percentage of nucleus internalization, and comparable cytotoxicity to cisplatin with an activity of 4.0 MBq/nmol [110,111]. Following the promising results obtained in vitro, the stability and the biodistribution profile of **20** through PET imaging was analyzed in vivo. It showed a promising tumor uptake ranging from 1.8 ± 0.4 to 3.0 ± 0.2% ID/g over 48 h. Furthermore, it demonstrated the ability to retard tumor growth with any observable signs of toxicity [112].

Another example is **21**, which was developed to target G-quadruplexes. Here, the Pt scaffold was functionalized with a ^111^In-DOTA moiety to assess its in vivo biodistribution via SPECT imaging [113]. Unfortunately, the complex exhibited limited cell permeability, highlighting the necessity for further research in this field (Figure 14).

Gallium-68 is a promising radioisotope for PET imaging, offering a suitable half-life (68 min) for rapid imaging and reduced patient exposure. Moreover, standard radiolabeling protocols are already established for this radioisotope. Notably, Sadler et al. conducted a pioneering in vivo imaging study using dynamic PET imaging to observe the whole-body distribution of a photoactivable Pt(IV) azido complex (**22**). The findings demonstrated a promising pharmacokinetic profile, indicating the potential for advancing ^68^Ga-DFO (DFO = deferoxamine) complexes in the image-guided treatment of therapeutic Pt(IV) prodrugs [114].

As a conclusion of this section, it needs to be highlighted that the design of novel theranostic platforms featuring both therapeutic (Pt-based complexes) and imaging modalities is still in its infancy and therefore further research needs to be carried out.

## 4. Magnetic Resonance Imaging (MRI) Contrast Agent-Pt Conjugates for Detecting and Treating Solid Tumors

MRI is indeed the most efficient technique for tumor imaging. It is non-invasive with excellent tissue contrast and spatial resolution (10–100 µm), and it is applicable in whole body with precise 3D localization. It employs radiofrequency radiation in the presence of carefully controlled magnetic fields, producing high-quality cross-sectional images of the body [115,116]. While the image resolution of MRI is superior to other tissue-penetrating imaging techniques such as PET or SPECT, its use is still limited in detecting disparities in the magnetic properties of tissues and organs in clinical practice [117].

**Figure 15:**
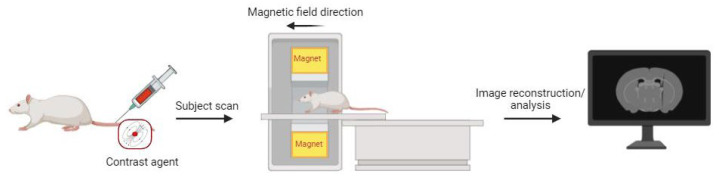
Representation of the basic principles of MRI (created with BioRender.com).

Contrast-enhanced MRI plays an increasingly key role in diagnostic medicine. Its usefulness originates from its capability to provide more detailed diagnostic information that cannot be obtained with other non-invasive techniques. The contrast agent is administered while the patient is in the scanner, and the diagnostic images appear within minutes (Figure 15).

The basis for an MRI signal is the precession of water hydrogen nuclei with respect to an applied magnetic field, and MRI contrast agents can be used to shorten the relaxation times of water molecules. They accumulate tissue-specifically and therefore enhance contrast in this specific organ, allowing for faster imaging (higher throughput) and for imaging that is less sensitive to artifacts caused by motion (better-quality images). Two different relaxation times are analyzed: longitudinal (spin–lattice relaxation time, T1) and the transversal (spin–spin relaxation time, T2). The rate constants corresponding to the T1 or T2 relaxation times are defined as 1/T1 and 1/T2. Contrast agents with higher relaxivities [r1 (=1/T1) and r2 (=1/T2)] give stronger contrast enhancement [118].

The contrast agents are small, such as hydrophilic gadolinium(III)-based chelates, but gadolinium is not the only element that can be used to generate MRI contrast. Indeed, iron oxide nanoparticles and manganese(II) complexes have been approved for liver imaging, although they are still not commercially successful. Paramagnetic elements such as gadolinium(III) and high–spin manganese(II) are for T1-weighted because of their shorter T1 time, while superparamagnetic contrast agents such as iron-oxide nanoparticles are for T2-weighted, for the same reason [119,120,121].

Gadolinium may also play an important role in therapeutic techniques, such as synchrotron stereotactic radiotherapy, in which the selective delivery of gadolinium to the cell nucleus would significantly enhance the efficacy of the treatment [122,123]. Gadolinium(III) complexes have also been explored as potential agents in an experimental anti-cancer treatment known as gadolinium neutron-capture therapy, which is closely related to the boron neutron-capture therapy [124,125]. When looking at the design of new small theranostic molecules, one common approach is that the therapeutic component (i.e., Pt-based scaffold) is covalently linked to the diagnostic component (i.e., Gd contrast agent) [117]. More recent studies showed the development of hybrid Gd-Pt theranostic agents incorporated into micelles [126,127] or nanoparticles instead [128,129]. All these approaches aim to mitigate the inherent cytotoxicity of the drug carrier [130].

### 4.1. Pt-Based Gd (III) Conjugates as Theranostic Contrast Agents

**Figure 16:**
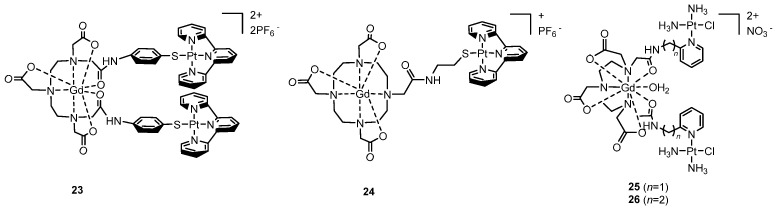
Structure of Pt (II)-based Gd-DTPA conjugate (**23**), Pt (II)-based Gd–DOTA conjugate (**24**) and Pt (II)-based Gd–DTPA complexes **25** and **26**.

In 2010, Crossley et al. [131] reported the pioneering work of gadolinium transport to a tumor cell nucleus via conjugation with a platinum complex. This innovative approach involved the functionalization of Gd–DTPA (DTPA = diethylenetriaminepentaacetic acid) with two Pt(II) (terpy) (terpy = 2,2′:6′,2″-terpyridine) units **23**. It was shown to function as a potent DNA intercalator of lung tumor cells. The tumor cells exhibited an enhanced accumulation capacity of both Pt and Gd with respect to the control, suggesting the presence of a tumor-selective uptake mechanism for the complex and the integrity of the complex in vitro. However, despite these observations, the Gd-Pt complex displayed significant cytotoxicity towards both normal and tumor cells, preventing its use as a potential theranostic agent (Figure 16).

Fenton et al. [132] presented a next generation of DNA metal-intercalators that addressed a few unfavorable key factors associated with the previous prototype. By replacing the acyclic DTPA ligand with the macrocycle DOTA (DOTA = 2,2′,2″,2‴-(1,4,7,10-tetraazacyclododecane-1,4,7,10-tetrayl)tetraacetic acid), the amide hydrolytic stability was enhanced. Moreover, by employing only a single Pt(II)-terpy unit, the cytotoxicity decreased with a concomitant increase in the cellular uptake while retaining an excellent DNA targeting ability (**24**).

Zhu et al. [133] developed two bifunctional Pt–Gd complexes (**25** and **26**) to monitor the temporal distribution of Pt drugs in vivo and to assess the therapeutic response in situ. These complexes partially dissociate in the tumor environment releasing the cytostatic Pt scaffold and the residual Gd moiety for imaging. The incorporation of Pt units significantly enhances the cellular uptake of Gd complexes, while the proton relaxivities of the Pt–Gd complexes surpassed those of Gd–DTPA under physiological conditions. Also, these complexes exhibit prolonged retention in mice kidneys, presenting an opportunity for non-invasive diagnosis of acute renal injury induced by nephrotoxic platinum drugs in mice models (Figure 16).

**Figure 17:**
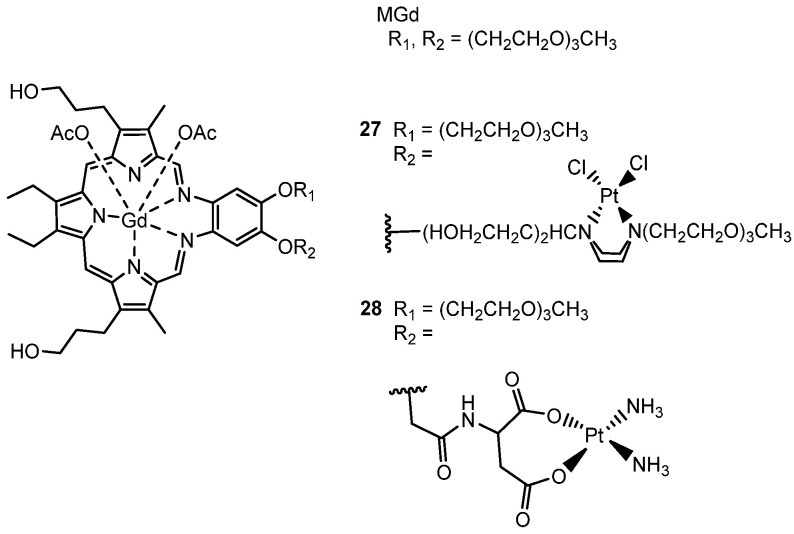
Structure of Motexafin-Gadolinium MGd and Pt (II)-based Motexafin-Gadolinium conjugates **27** and **28**.

A different approach was developed through the conjugation of a gadolinium(III) texaphyrin to platinum-based scaffolds as a strategy for selective targeting tumoral tissues and overcoming mechanisms associated with cisplatin resistance. Metallotexaphyrins, a class of expanded porphyrins (e.g., motexafin gadolinium [MGd]), demonstrated notable accumulation in both primary and metastatic tumors [134,135] and possess intrinsic anticancer activity attributed to their macrocyclic ligand-centered redox activity [136,137]. Since 2004, Sessler’s group has focused its interest on the development of texaphyrin drug conjugates as promising candidates for tumor localization [138,139,140,141,142,143] and on the combination of Motexafin gadolinium drug (MGd, Figure 17) to mediate the cancer-specific reduction of Pt(IV) agents [144]. Magda et al. [138] reported the first generation of texaphyrin-type drugs (**27** and **28**), where established anticancer agents such as cisplatin were covalently conjugated to a tumor-localizing texaphyrin core. Unfortunately, this initial study faced limitations in in vitro evaluation due to poor solubility and hydrolytic instability (Figure 17).

**Figure 18:**
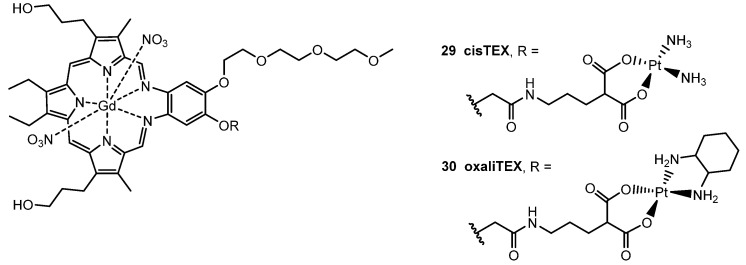
Structure of Pt (II)-based Texaphyrinmalonate conjugates cisTEX (**29**) and oxaliTEX (**30**).

More recently, a novel gadolinium texaphyrinmalonate-Pt conjugate **29** was reported by Arambula et al. [139], which showed anti-proliferative activity in vitro comparable to that of carboplatin. The inclusion of the malonate chelating group, analogous to that present in carboplatin, was strategically designed to facilitate Pt release under physiological conditions (Figure 18). Furthermore, working with an ovarian cancer cell model, an enhanced intracellular platinum accumulation was observed, as well as a greater number of Pt–DNA adducts compared to the controls (i.e., methilmalonatoplatinum, carboplatin). Despite these findings, the DNA damage repair induced by the conjugate remained comparable to that of cisplatin, suggesting the persistence of other molecular mechanisms of resistance, such as failure to reactivate the tumor suppressor factor p53 [140].

To overcome key platinum pharmacological and molecular resistance mechanisms in vitro, a novel texaphyrin-oxaliplatin-like conjugate **30** was synthesized and biologically screened (Figure 18). While very effective in terms of anticancer activity, it was observed that the Pt moieties were hydrolytically unstable in aqueous environments, resulting in slow, uncontrolled release of the Pt(II) core. Therefore, difficulties were encountered in terms of formulating this generation conjugate for in vivo applications [141].

**Figure 19:**
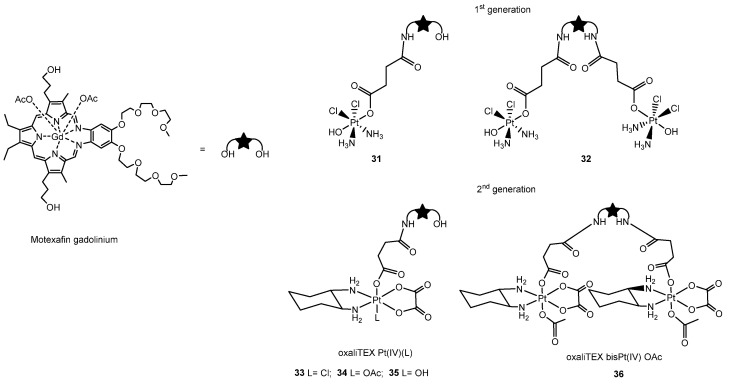
Structure of first generation of Pt (II)-based texaphyrinmalonate conjugates **31** and **32** and second generation of Pt (IV)-based texaphyrin **33**–**36**.

In order to develop a targeting Pt-texaphyrin system capable of controlled Pt release, a second generation of platinum(IV)-texaphyrin conjugates was synthetized later by the same group. The presence of a Pt(IV) core increased the hydrolytic stability relative to the first-generation Pt(II) systems, and Pt(IV) conjugates were able to release the Pt(II) scaffold in a controlled fashion upon exposure to light or a reducing environment. The asymmetric Pt(IV) based on cisplatin scaffold was chosen as the platinum precursor for **31** and **32** (Figure 19, first-generation). Both conjugates demonstrated a very good anti-proliferative activity in vitro against both wild-type and cisplatin-resistant ovarian cancer cell lines, showing improvements in the IC_50_ values for both cell lines, as well a decrease in the resistance factor (the resistance factor is calculated as the ratio between the IC_50_ values of cisplatin-resistant ovarian cancer and wild-type cell lines) [142].

A combination of both in vitro and in vivo studies on the latest generation of Pt(IV)-based texaphyrin conjugates (**33**–**36** Figure 19) showed promising results in overcoming platinum-resistant ovarian and colon cancers. In this case, the 1,2-diaminocyclohexane (DACH) ligand environment around the platinum center (oxalilplatin scaffold) is believed to be crucial in producing agents capable of overcoming p53-based platinum resistance. Texaphyrin conjugates based on a Pt(IV)–DACH complex were designed to deliver an active Pt(II), analogue to oxaliplatin, in the reducing environments characteristic of many solid tumors and to overcome common p53-related cisplatin resistance mechanisms. Among the four different complexes shown in Figure 19, **34** demonstrated a superior efficacy in retarding/inhibiting tumor growth in mouse models, including those with clinically relevant Pt-resistant mouse models, outperforming the most closely related current platinum-based standards of care. These findings suggest that this complex could play a promising role in clinical applications [143].

**Figure 20:**
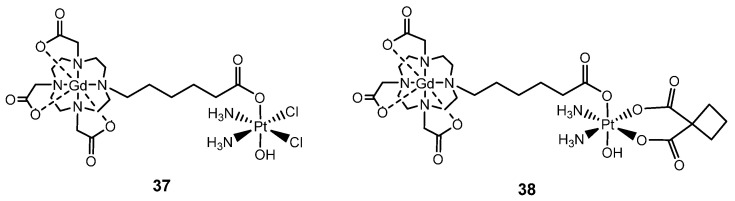
Structure of Pt (IV)-based Gd (III) conjugates as theranostic agents with Gd(III) MRI contrast agent in axial position of cisplatin **37** and carboplatin **38**.

Inspired by this promising work of Sessler and coworkers, who demonstrated how Gd(III) complexes can be used synergistically with Pt(IV) prodrugs, Adams et al. took the opportunity to investigate Gd(III)–Pt(IV) mixed-metal complexes as theranostic agents [145,146]. Firstly, two Gd(III)–Pt(IV) conjugates (**37** and **38**, Figure 20) were synthesized by coupling a Gd(III) MRI contrast agent in the axial position of a cisplatin and carboplatin-based scaffold forming novel Pt(IV) complexes [145]. Unlike the typical Gd(III) contrast agents, which are confined to the extracellular space surrounding tumors, these complexes are tailored for intracellular contrast enhancement of cancer cells due to the presence of the more lipophilic platinum moiety. Among the two agents, **37** emerged as the most promising, displaying greater cellular toxicity, higher intracellular accumulation of Gd(III), and superior MR contrast enhancement in vitro. Cellular accumulation analyses between in vitro and in vivo studies highlighted the variations in MR contrast enhancement, offering a potential strategy for imaging Pt in resistance cell lines. This platform could potentially be applied across various chemotherapeutics, particularly in cases where decreased drug accumulation is a dominant mechanism of chemoresistance [146].

### 4.2. Multifunctional Nanodevices Combining Platinum Complexes and Magnetic Resonance (MRI)

Nanotechnology is one of the fast-moving areas in the medicinal field and it contributes significantly to the progress of medicinal science, with an increasing role in diagnostics, in vivo imaging, and improved treatment of disease [146,147]. The usefulness of nanoparticles is mainly derived from their small size and large surface area for in vivo drug delivery [148]. Nanoscale carriers as drug delivery systems can maximize the therapeutic efficacy and minimize the side effects of loaded drugs. Indeed, they can be accumulated selectively in solid tumors exploiting the enhanced permeability and retention effect [149,150,151]. Engineered nanodevices can target cancer cells, release their cargo in response to a stimulus, and selectively deliver drugs to the final target, and this approach can enhance the pharmacological activity of the drugs and improve the pharmacokinetics [152,153,154]. In oncology, nanotechnology finds application as nanoscale Trojan horses to bypass drug inactivation pathways in the cytoplasm and deliver drugs efficiently to the target nuclei, overcoming multidrug resistance in cancer cells [155]. In the last decade, several families of nanoparticle therapeutics platforms have been developed to selectively deliver Pt-based anticancer drugs to tumor sites, including inorganic nanoparticles [156,157] and polymeric micelles [118,158].

Recent advances in nanomedicine include the functionalization of the surface of nanodevices with targeting ligands [159], as well as imaging and therapeutic moieties [160], or the loading of drugs with an integration of various imaging elements that allows for multimodal and multifunctional nano-agents.

MRI stands out as one of the most extensively utilized imaging techniques for nanostructures, as MRI contrast agents are mainly paramagnetic complexes or magnetic nanoparticles. As previously mentioned, the direct conjugation of imaging contrast agents to therapeutic entities, such as Pt-complexes, has been found to compromise both the biodistribution and biological activity of the drug [138,139,140,141]. Nevertheless, the integration of imaging functionality into nanoscale drug carriers preserved these important attributes while potentially enhancing the targeting properties towards diseased sites and improving both diagnostic and therapeutic effectiveness [161,162]. Specific examples of how nanodevices have been exploited to enhance T1 and T2 contrast agents are described in Section 4.3 and Section 4.4.

### 4.3. T1-Enhanced Contrast Agents

Regarding T1-enhanced contrast agents like paramagnetic molecules, such as gadolinium and manganese, higher relaxivity gives a stronger contrast enhancement. A strategy aimed at enhancing the relaxivity of Gd contrast agents involves reducing the mobility of the metal complex. This is achieved by conjugation/loading them onto nanoscale platforms such as micelles, thereby slowing molecular reorientation [163,164]. Polymeric micelles have been considered one of the most promising drug delivery systems in the field of cancer therapy and have revealed a reduction in side effects and high effectiveness against various intractable tumors. The development of micelles with both imaging and therapeutic functions has enabled real-time visualization of their distribution inside the body, facilitating the optimization of treatment protocols [160,165,166].

**Figure 21:**
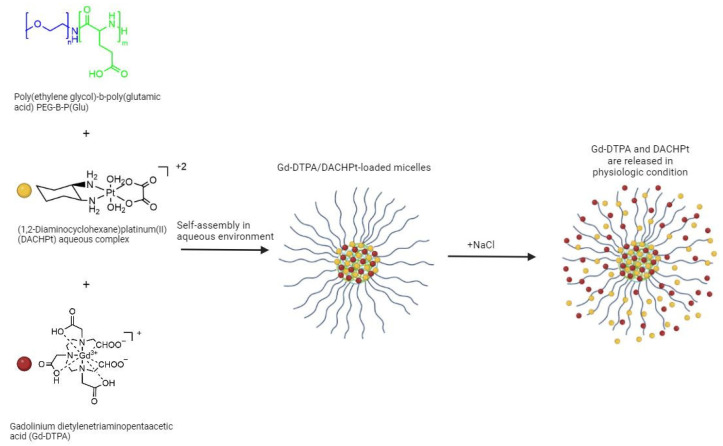
Synthetic view of the structure of Gd–DTPA/DACHPt-loaded micelles and the consequently releasing of Pt and Gd complexes from the micelles (created with BioRender.com).

Kaida et al. [127] developed a self-assembly polymeric micelle that integrates both MRI functionality and cancer therapeutic capacity (Figure 21). This was achieved by incorporating gadolinium-diethylenetriaminepentaacetic acid (Gd–DTPA) as a contrast agent and the complex (1,2-diaminocyclohexane)platinum(II) (DACHPt), based on the DTPA anticancer drug oxaliplatin scaffold. In vitro and in vivo studies on pancreatic tumor models showed an enhanced tumor accumulation of the micelles, along with improved drug release, leading to enhanced efficacy and decreased toxicity. Additionally, real-time monitoring of drug distribution and tracking of tumor size were achieved. Gd–DTPA/DACHPt-loaded micelles also revealed an outstanding contrast enhancement attributed to the accumulation and to the increase in the relaxivity, suggesting a great potential of this modality for the clear detection of the tumor. In a more recent study [167], the same group examined the feasibility of using these Gd–DTPA/DACHPt-loaded micelles to simultaneously diagnose and treat a clinically rat model of hepatocellular carcinoma (HCC) and demonstrated the safety and the enhanced efficacy of these micelles for the selective imaging and treatment of HCC.

**Figure 22:**
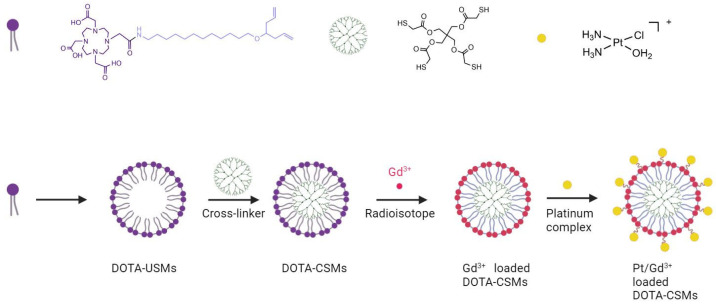
Schematic procedure of Pt/Gd^3+^-loaded DOTA micelles (created with BioRender.com).

An alternative approach was described by Feng et al. [126] with the conjugation of Gd(III) and cisplatin on the surface of DOTA functionalized cross-linked small molecular micelles, as seen in Figure 22. The in vivo evaluation showed that the new theranostic nanoplatform exhibited not only a stronger and longer-lasting contrast enhancement efficacy but also a greater tumor growth inhibition rate with lower side effects. Due to the toxicity associated with the use of paramagnetic chelates for T1-weighted MRI, there is a demanding need to explore alternative transition-metal oxides [168].

**Figure 23:**
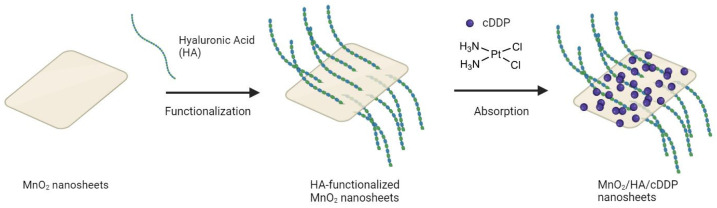
Schematic illustration of preparation of MnO_2_/HA/cDDP nanosheets (created with BioRender.com).

More recently, manganese dioxide (MnO_2_) nanostructures have emerged as a novel responsive MRI-T1 contrast agent [169]. In these nanoparticles, manganese presents a 4+ oxidation state, displaying weak paramagnetism. However, in the presence of biologically relevant concentrations of GSH or other redox-active species, MnO_2_ nanostructures undergo reduction to form free aqueous Mn(II) and O_2_. Mn in its 2+ oxidation state has two unpaired electrons and exhibits strong paramagnetism, leading to an enhanced MRI signal [170]. In this context, Hao et al. [169] reported a very promising approach by developing a tumor-targeted theranostic delivery system comprising degradable MnO_2_ nanosheets functionalized with hyaluronic acid (HA) and loaded with cisplatin, as shown in Figure 23. Here, HA was used as a delivery molecule, as overexpression of its receptors has been found in various tumors. Once the tumor is reached, controlled drug release is achieved by the tumor-microenvironment-responsive degradation of MnO_2_ nanosheets.

However, the preparation of these theranostic agents required challenging multistep reactions. A few years later, Brito et al. [171] described a MnO_2_–Pt (IV) nanostructure synthesized through a simplified one-pot facile ultrasonication reaction, significantly reducing the time and complexity of the materials. These nanoparticles demonstrated a robust off/on MRI behavior both in vitro and in vivo in response to the reducing agents. They also exhibited efficiency in inducing cell death, comparable to that of cisplatin. However, further systemic imaging and therapeutic studies are still required to fully assess their potential.

**Figure 24:**
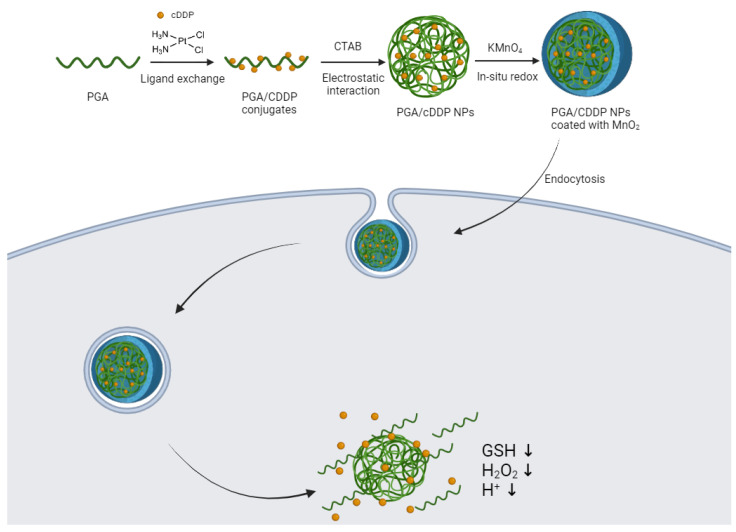
Schematic diagram of the preparation process and controlled drug release of PGA/CDDP@MnO_2_ NPs (created with BioRender.com).

In the same year, Zhang et al. [172] presented a promising strategy aimed at overcoming the primary limitation associated with polymeric micelles as drug delivery vehicles such as premature release, and drug binding with plasma proteins before reaching tumor cells. They developed poly(glycolic acid)/cisplatin (PGA/CDDP) NPs via electrostatic interaction, which were further coated with MnO_2_ to prevent premature leakage and augment the therapeutic effect of cisplatin in tumor cells. Additionally, the MnO_2_ shells can react with high concentration of GSH, H_2_O_2_ and H^+^ for a multi-responsive controlled drug release (Figure 24). This innovative approach led to a significant enhancement in the toxic effect of pure cisplatin of approximately 2–3 times, upon coupling with MnO_2_. Furthermore, serving as a multi-functional nanoplatform, PGA/CDDP@MnO_2_ NPs holds considerable promise for imaging and diagnosing various tumors.

### 4.4. T2-Enhanced Contrast Agents

The most studied T2-enhanced contrast agents are superparamagnetic iron-oxide (Fe_3_O_4_) nanoparticles. In this case, the value of T2 is inversely proportional to its cross-sectional area, meaning that the same amount of magnetized material is more effective when dispersed as fewer large aggregates rather than numerous smaller ones [173]. Mn-doped Fe_3_O_4_ nanoparticles may be used as simultaneous dual-modal probes for T1/T2 MRI, providing complementary imaging information for an early and precise diagnosis [174].

**Figure 25:**
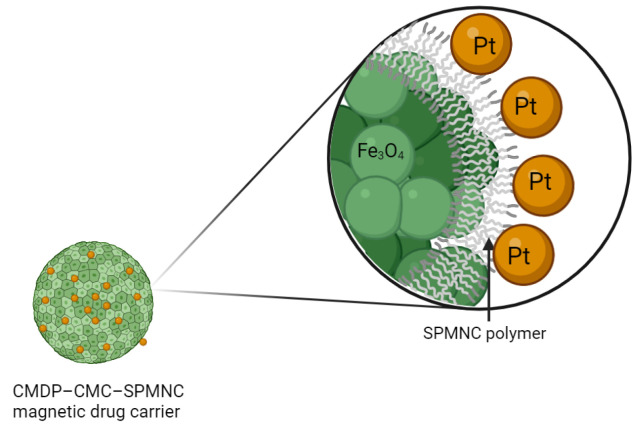
Structure of conjugate CMDP–CMC–SPMNC magnetic drug carrier (created with BioRender.com).

SPIONs (Super Paramagnetic Iron Oxide Nanoparticles) possess some excellent properties, such as biocompatibility, hydrophilicity, nontoxicity, and no immunogenicity and as a result, they have garnered significant attention in recent years as both drug carriers and diagnostic agents [175,176]. Based on this idea, Xing et al. [177] synthesized superparamagnetic magnetite nanocrystal clusters. Sodium carboxymethylcellulose (Na-CMC SPMNC) was chosen as a biocompatible and biodegradable polymer for external coating. The platinum drug (monochlorinated CDDP, CMDP) was loaded onto the clusters, forming a conjugate of CMDP–CMC–SPMNC magnetic drug carriers that were able to transport platinum drug preferentially to its biological target by making use of a magnetic field (Figure 25). In addition to the benefits of simplicity in preparation and high loading capacity, the system significantly enhances the cellular uptake of platinum drugs. The cytotoxicity towards the human cervical cancer HeLa cells and the human hepatocarcinoma HepG2 cells of the drug carrier conjugate are higher than or at least comparable to those of cisplatin.

In subsequent work, Wang et al. [128] reported a study where an analog of cisplatin (CMDP) was tethered to modified maghemite nanoparticles (OTPBA–SPION) for targeted cancer theranostics under the influence of an external magnetic field. This approach offered several advantages, including particle size conducive to tumor cell entry, straight-forward preparation, high drug-loading capacity, nontoxic degradation of the core, and more importantly, and specific accumulation in cancer tissues under an external magnetic field. However, there was a concern that Pt(II) moieties could react during delivery, potentially posing toxic effects on normal tissues.

To overcome these limitations, Zhu et al. [178] proposed a novel strategy involving the substitution of the previous Pt(II) pharmacophore with a Pt(IV) prodrug complex that is more inert and more stable than Pt(II) complexes as described earlier, thereby mitigating undesirable side reactions in the blood plasma. In this study, SPIONs were retained to construct a targeted Pt prodrug system, with polyethylene glycol (PEG) coating applied to enhance in vivo stability. A functionalized prodrug of cisplatin (HSPt) was loaded onto the surface of the PEGylated SPIONs. The system produced a significant negative contrast in MRI, suggesting its potential as a theranostic agent for chemotherapy. Cytotoxicity was enhanced in the presence of GSH, facilitating controlled drug release within the tumor microenvironment.

**Figure 26:**
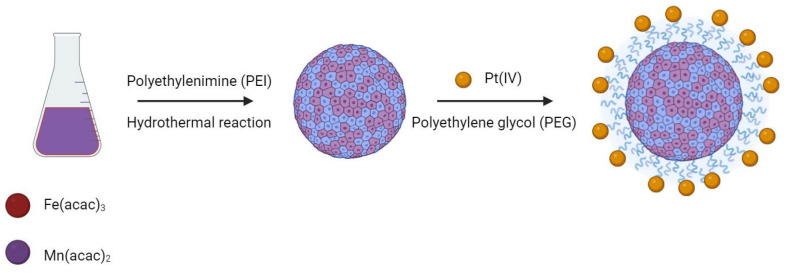
Scheme of the preparation of magnetic Pt-FMO nanoparticles (created with BioRender.com).

Cheng et al. [174] designed and developed an innovative Pt-FMO nanoplatform (where FMO stands for manganese deposited iron oxide, Fe–Mg composite oxide) that served as a simultaneous dual-modal probe for T1/T2 MRI while also enabling mutual beneficial cascade reactions to promote ferroptosis and apoptosis in combination with its utilization as an MRI agent. A cisplatin prodrug Pt(IV) containing a carboxylic acid in the axial position was covalently conjugated to the surface of FMO via amide bond formation and was activated through endogenous GSH to generate the Pt(II) drug (Figure 26). In vitro and in vivo essays demonstrate that a remarkably lower Pt dose (8.89%) of Pt-FMO was able to achieve a significant in vivo antitumor effect of cisplatin. The combination effect on tumor cells through cisplatin-induced apoptosis and Fenton-reaction-promoted ferroptosis is crucial to achieve such promising results.

## 5. Conclusions

The overview provided in this review about recent advancements in the use of Pt-based drugs coupled with various imaging techniques showed a huge potential in future translation to clinical studies. Nevertheless, further studies are needed to overcome the limitations of the different imaging techniques involved and to improve the activity of the anticancer agent.

Regarding fluorescence imaging, coupling Pt compounds with fluorophores to study their spatial-temporal distribution in cells poses a challenge because the fluorophore’s structure can alter the original Pt-based compound’s physicochemical properties. To address this issue, various exogenous turn-on probes have been developed. Among the different NIR-fluorescent probes mentioned before, AuNCs have the highest potential for in vivo tumor imaging due to their small size, tunable emissions from the visible to the NIR range, excellent biocompatibility, easy surface functionalization with peptides/proteins/antibodies for active cell-targeting, and enhanced EPR effect.

Regarding SPECT imaging, CISSPECT^®^ was the most efficient theranostic agent developed to predict chemotherapy treatment efficacy of cisplatin, external radio beam therapy optimization and reduction in side effects such as severe kidney toxicity. While CISSPECT^®^ shows promising results, PET imaging offers distinct advantages, including higher sensitivity, superior resolution, and the use of shorter-half-life radiotracers that reduce radiation exposure. Notably, we highlight the promising outcomes of terpyridine platinum-based complexes conjugated with ^64^Cu-NOTA as an innovative theranostic platform aimed at overcoming platinum drug resistance. Although PET imaging of Pt-theranostic compounds is still a rare technique, recent developments, such as the ^68^Ga-Pt(IV)azido complex (**22**), demonstrate significant potential for image-guided treatment of therapeutic Pt(IV) prodrugs.

In the field of MRI imaging, significant efforts have been made in the development of Pt-Gd complexes. While first-generation Gd-Pt complexes encountered challenges such as cytotoxicity and stability concerns, ongoing research has led to substantial advancements in their design and efficacy. Particularly noteworthy are texaphyrin-based and Pt(IV) conjugates, which hold promise for overcoming platinum resistance through targeted therapeutic action. Notably, complex **34** has demonstrated superior efficacy in inhibiting tumor growth, underscoring its potential for clinical applications. Moreover, nanotechnology has highly promoted MRI contrast agents research, thus offering versatile platforms that decrease the toxicity of Gd-based probes and achieve a stronger contrast enhancement. However, we believe SPIONs have a huge potential as both MRI contrast agents and drug delivery platforms due to their high biocompatibility, low toxicity, decreased immunogenicity, extended imaging windows, and easy surface functionalization with ligands or therapeutic agents.

For the future of molecular medicine, developing dual-modality molecular imaging probes will be pivotal, as no single imaging modality can provide all the necessary information. For example, PET offers excellent sensitivity and metabolic functionality but suffers from poor spatial resolution. Conversely, MRI provides supremely high-resolution anatomic information in the sub-millimeter range. By synergistically combining these modalities, we can achieve comprehensive diagnostic capabilities. Several current strategies are already moving in this direction [179,180].

Therefore, our future contribution in this field will be to develop SPION-based platforms with Pt-derivatives coupled to radio-chelators to create MRI/PET imaging theranostic probes for both cancer diagnosis and treatment. We aim to evaluate these probes both in vitro and in vivo to assess their potential for clinical translation.

In conclusion, the use of Pt-based complexes in a theranostic scenario emerges as a strategy to solve current issues of one of the widest-used anticancer drugs and to design an individual therapeutic intervention in combination with an appropriate diagnostic methodology. We are confident that this direction is a promising approach so that personalized molecular medicine may soon become a clinical reality.

## Figures and Tables

**Table 1:** Molecular mechanisms of cisplatin resistance.

	Molecular Mechanism	Factor (Function)	Preclinical Evidence	Clinical Evidence
Pre-target resistance	Reduced uptake	CTR1 (plasma membrane copper transporter)	Knockdown of CTR1 reduces cisplatin uptake (80%) in both yeast and mouse embryonic fibroblasts [15].	NSCLC tumors with undetectable CTR1 expression exhibit reduced intratumorally platinum concentration [5].
	Increased efflux	ATP7A/ATP7B (copper P-type ATPases involved in the regulation of ion homeostasis).	Overexpression of ATP7B in human oral squamous cells resulted in cisplatin resistance [16]. Human ovarian carcinoma cells with ATP7A overexpression show higher resistance to cisplatin, carboplatin and oxaliplatin [8].	Elevated expression of ATP7A is associated with reduced clinical outcome for patients with ovarian cancer [7]. High ATP7B expression in ovarian carcinoma correlates with poor clinical outcomes in cisplatin-based chemotherapy-treated patients [17].
	Increased inactivation	GSH/γ-GCS/GST	Cisplatin resistant cells often exhibit elevated levels of GSH, γ-GCS and GST [9,10,11].	No conclusive clinical evidence.
On-target resistance	Nucleotide excision repair (NER)	ERCC1 (single-strand endonuclease)	Increased ERCC1 expression is associated with cisplatin resistance in human hepatocellular carcinoma and human cervical tumor cell lines [18,19].	Positive ERCC1 expression and cisplatin clinical resistance in non-small cell lung, ovarian cancer and human hepatocellular carcinoma [18,20,21].
	DNA mismatch repair (MMR)	MLH1 (protein that initiates DNA repair)	–	MLH1 deficiency is correlated with cisplatin resistance in esophageal cancer [22]. Low MLH1 expression was associated with improved prognosis in ovarian cancer [23].
	NER, MMR and base excision repair	HMGB1 (protein involved in transcriptional regulation, DNA replication and repair)	Higher HMGB1 expression was associated with higher cisplatin resistance in lung adenocarcinoma cell line [24]. HMGB1-induced cell autophagy contributes to cisplatin resistance in cervical cancer cells [25].	HMGB1 overexpression significantly promoted cisplatin resistance of NSCLC in vitro and in vivo [26]. Positive HMGB1 expression is strongly associated with cisplatin resistance in gastric cancer patients [27].
Post-target resistance	–	TP53 (tumor-suppressive protein that controls DNA repair and apoptosis in response to stress).	Full knock-out of TP53 in human testicular cancer-derived embryonal carcinoma cell line resulted in higher cisplatin resistance [28,29].	NSCLC patients harboring wild-type TP53 are associated with longer survival after cisplatin-based chemotherapy than patients with TP53 mutations [30].
Off-target resistance	Autophagy	–	Ovarian and lung cancer cells showed cisplatin-induced autophagy [31,32,33].	No data reported.
	–	ERBB2/HER-2 (Oncogenic EGKR-like receptor that is overactivated in different types of cancer).	HER2 protein is overexpressed in cisplatin-resistant gastric cancer cells [34].	High expression of HER2 is correlated with cisplatin resistance in NSCLC patients [35].

**Table 2:** Example of imaging techniques used in theranostics [45,46].

Imaging Method	Imaging Time	Main Purpose	Benefits	Limitations
Fluorescence	Sec-Min	Monitoring tumor response to treatment, metastatic spread; imaging gene expression and protein–protein interaction.	Relativity non-invasive and cheap. Easy labeling with many fluorescent molecules available. High sensitivity. No ionizing radiation.	Limited clinical applications. Potential incompatibility and toxicity of fluorescent probes. Limited wavelength range (700–900 nm). Limited tissue penetration (≤2 cm).
PET	Min	Pharmacodynamics, pathophysiology, pharmacokinetics.	High sensitivity. Functional imaging is feasible. Fully quantitative.	Poor spatial resolution. Requires use of ionizing radiation. Limited accessibility.
SPECT	Min	Pharmacodynamics, pathophysiology, pharmacokinetics	High sensitivity. Quantitative. High versatility.	Requires use of ionizing radiation. Poor spatial resolution.
MRI	Min-hours	Pharmacodynamics, pathophysiology, pharmacokinetics, anatomy.	Good spatial resolution. No ionizing radiation. Several contrast agents and nanodevices are widely used for clinical imaging.	Poor sensitivity. Difficult real-time imaging. High dosage of contrast agent (potential toxicity and accumulation).

**Table 4:** Characteristics of main Pt radionuclides.

Radionuclide	Half-Life (Days)	Energies (keV)	Decay Mode	Method of Production
^191^Pt	2.86	539	EC ^1^	^191^Ir (d,2n)^191^Pt
^193m^Pt	4.33	66–77	IT ^2^ (100%) Auger electrons.	^193^Ir(d,2n)^193m^Pt
^195m^Pt	4.02	66–77, 99, 129	IT (100%) Auger electrons.	^194^Pt(n,γ)
^197^Pt	0.83	77, 191	β^−^	^196^Pt(n,γ)

^1^ EC = electron capture. ^2^ IT = isomeric transition.

**Table 5:** Selected radionuclides used in PET and SPECT, their production mode and decay properties [106].

Radionuclide	Half-Life	E_max_ (keV)	Radiation	Production	Production Reaction
PET radionuclides					
^13^N	10 min	1199	β+ (100%)	Cyclotron	^16^O(p,α)^13^N
^18^F	110 min	634	β+ (97%)	Cyclotron	^18^O(p,n)^18^F
^64^Cu	12.8 h	656	β+ (18%)	Cyclotron	^64^Ni(p,n)^64^Cu
^68^Ga	67.6 min	1900	β+ (89%)	Generator	^68^Ge → ^68^Ga + β^−^
SPECT radionuclides					
^99m^Tc	6.0 h	141	γ	Generator	^99^Mo → ^99m^Tc
^111^In	67.9 h	245, 172	γ	Cyclotron	^112^Cd (p, 2n)^111^In

## Data Availability

Data sharing is not applicable.
